# Imbalance of heterologous protein folding and disulfide bond formation rates yields runaway oxidative stress

**DOI:** 10.1186/1741-7007-10-16

**Published:** 2012-03-01

**Authors:** Keith EJ Tyo, Zihe Liu, Dina Petranovic, Jens Nielsen

**Affiliations:** 1 Department of Chemical and Biological Engineering, Chalmers University of Technology, Kemivägen 10, SE-41296 Göteborg, Sweden; 2 Department of Chemical and Biological Engineering, Northwestern University, 2145 Sheridan Rd. Tech E136, Evanston, IL 60208, USA

**Keywords:** Protein secretion, unfolded protein response, *HAC1*, protein production, oxidative stress

## Abstract

**Background:**

The protein secretory pathway must process a wide assortment of native proteins for eukaryotic cells to function. As well, recombinant protein secretion is used extensively to produce many biologics and industrial enzymes. Therefore, secretory pathway dysfunction can be highly detrimental to the cell and can drastically inhibit product titers in biochemical production. Because the secretory pathway is a highly-integrated, multi-organelle system, dysfunction can happen at many levels and dissecting the root cause can be challenging. In this study, we apply a systems biology approach to analyze secretory pathway dysfunctions resulting from heterologous production of a small protein (insulin precursor) or a larger protein (α-amylase).

**Results:**

*HAC1*-dependent and independent dysfunctions and cellular responses were apparent across multiple datasets. In particular, processes involving (a) degradation of protein/recycling amino acids, (b) overall transcription/translation repression, and (c) oxidative stress were broadly associated with secretory stress.

**Conclusions:**

Apparent runaway oxidative stress due to radical production observed here and elsewhere can be explained by a futile cycle of disulfide formation and breaking that consumes reduced glutathione and produces reactive oxygen species. The futile cycle is dominating when protein folding rates are low relative to disulfide bond formation rates. While not strictly conclusive with the present data, this insight does provide a molecular interpretation to an, until now, largely empirical understanding of optimizing heterologous protein secretion. This molecular insight has direct implications on engineering a broad range of recombinant proteins for secretion and provides potential hypotheses for the root causes of several secretory-associated diseases.

## Background

The protein secretory pathway is an extensive process in eukaryal cells, as it is responsible for processing approximately one-third of all proteins. Substantial cellular resources are therefore utilized to maintain this pathway's functions, and stressed conditions in the secretory pathway have consequences for the whole cell [1]. Distress in secretory pathway organelles has been implicated as the molecular basis for several diseases, for example, β cell apoptosis in diabetes, cystic fibrosis, and prion-related disease, among others [2]. In biotechnology, efficient secretion of useful recombinant proteins in yeast and fungi is a key industrial objective with applications in enzyme production required for the production of biofuels, detergents, fabrics, food, and biologics, such as imunoglobulins, hormones, and vaccines. Significant effort has gone into engineering yeast for increasing protein secretion [3]. Strategies, such as changing environmental parameters (for example, temperature, media composition) [4] or altering genetics, can increase secretion for some proteins, but they rarely represent generic solutions for improving protein secretion [5,6]. The lack of a single engineering strategy that improves protein secretion across the board implies that there are several possible bottlenecks in the secretory pathway, and different proteins may be constrained in different ways. There is therefore a requirement for more fundamental insight into this complex pathway that involves a very large number of components.

In yeast, the secretory pathway is a multi-organelle system that is responsible for trafficking proteins to the extracellular space, cell membrane, or vacuole [7]. During this transit, multiple processes must be coordinated, including folding, specific proteolytic cleavage, glycosylation, and disulfide bond formation, all with a layer of quality control at key check points. The pathway requires substantial cellular resources to perform these tasks, such as glycans, electron acceptors, electron donors, and ATP. In the ER, the nascent peptide is folded into its native structure while disulfide bonds are formed. The rate of protein folding is dependent upon the complexity of the protein to be folded, the availability of chaperones to assist folding, and ATP used by the chaperones [1]. Proteins that are slow to fold or terminally misfolded proteins are removed from the ER via the ER-associated degradation (ERAD) pathway [8]. Disulfide bond formation requires the removal of electrons from cysteine thiols via protein disulfide isomerase (PDI) and Ero1p to the final electron acceptor, typically oxygen [9,10]. This process produces reactive oxygen species (ROS) in stoichiometric amounts to the number of disulfide bonds formed [11]. Disulfide bond formation is random, and incorrect bond pairs must be exchanged for native bonds via PDI-based processes [12]. In addition, reduced glutathione (GSH) acts as a buffer for the redox state of the ER [13]. A more detailed description of oxidative protein folding can be found in the reviews by Sevier *et al. *and Chakravarthi *et al. *[14,15].

The secretory pathway must adjust the chaperone capacity, oxidizing equivalents, ATP, glycan, and other metabolic requirements, as well as trafficking patterns, based on the portfolio of proteins that need to be expressed at a given time, and the resources required to process that set of proteins. In yeast, the unfolded protein response (UPR) is one transcriptional mechanism that adjusts secretory resources and controls to handle overload of the folding machinery in the ER [16]. In the UPR, accumulation of unfolded proteins in the ER signals a pathway that results in translation of Hac1p, a transcription factor (TF) known to activate or repress over 100 genes, including many ER-associated proteins such as Kar2p, Pdi1p, and Ero1p [17].

In this study, we identified biological mechanisms which alter the secretory pathway in response to secretion of recombinant proteins with different properties (size, number of disulfide bonds, and glycans) in a Hac1p-dependent and independent manner. The secretory pathway was perturbed by secreting a small protein, human insulin precursor (IP), or a comparatively larger protein, α-amylase, in wild-type (WT) and *Δhac1 Saccharomyces cerevisiae*. These proteins were chosen because the two proteins elicit different behavior in the secretory pathway. These differences will arise because α-amylase is a relatively larger (and likely more difficult to fold), has an odd number of cysteines (which may complicate disulfide isomerization) and has glycosylation, compared to insulin which is small, has even number of cysteines, and is not glycosylated. As well, α-amylase has one more disulfide bond than IP. To identify biological mechanisms, we characterized changes in physiological properties (specific growth rate, carbon utilization efficiency, and recombinant protein secretion), TF activity (as inferred from transcriptome analysis) and metabolic demand (as inferred by changes in metabolic flux diversion). Through this, we identified the following biological processes: amino acids recycling from degraded proteins, trans-Golgi network (TGN) sorting changes, overall expression repression, and oxidative stress. Motivated by secretory-related oxidative stress observations, we present a model for disulfide bond formation and electron transfer in the ER which takes into account thermodynamic irreversibilities caused by differences in electron affinity. The proposed model explains the non-stoichiometric ROS formation that we observed that results from disulfide bond formation and causes oxidative stress under folding-stress conditions. If proven by genetic and biochemical results, the futile cycle model yields insight into a fundamental problem in secretory stress and reveals new avenues to reduce oxidative stress and increase productivity in industrial protein production.

## Results

### Protein size and Hac1p activity affect protein secretion quantity and cell growth

Yeast strains were constructed that produce and secrete (a) IP or (b) α-amylase and were compared to yeast strains containing (c) an empty vector in both wild-type and *HAC1 *deletion backgrounds. IP and α-amylase were chosen because they are very different types of proteins to secrete. IP is 51 amino acids in length, with six cysteines forming three disulfide bonds, and no glycosylation. α-amylase is 478 amino acid in length, with nine cysteines forming only four disulfide bonds and one glycosylation. The odd number of cysteines in α-amylase complicates disulfide pairing, as the random isomerization process may incorporate the cysteine that should not be incorporated into a disulfide bond. Both proteins were targeted for secretion using a *YAP3 *pre sequence (21 amino acids, cleaved off in the ER) and a rationally designed pro sequence (TA57, 42 amino acids, no glycosylation or disulfides) were cloned behind a *TDH3 *promoter in a high copy 2 micron plasmid [18]. α-amylase was expressed using the same plasmid, promoter, and leader sequences. These strains are named WN (WT with empty vector), WI (WT secreting IP), WA (WT secreting α-amylase), dN (*Δhac1 *with empty vector), dI (*Δhac1 *secreting IP), and dA (*Δhac1 *secreting α-amylase). Strains were characterized in batch fermentation to understand the effects on cell physiology.

**Figure 1:**
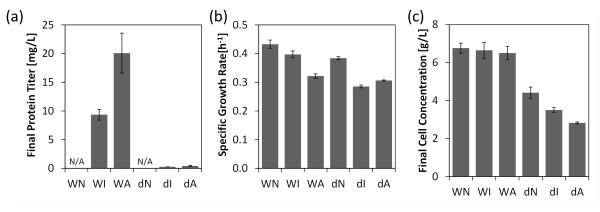
**Secretory perturbations affect yeast physiology**. **(a) **Final recombinant protein titer. *Δhac1 *strains were severely inhibited in recombinant secretion. **(b) **Specific growth rate on glucose. The combination of *Δhac1 *and recombinant secretion had the most severe effect on growth, however even in wild-type background, α-amylase hindered growth. **(c) **Final cell concentration. Wild-type, no protein secretion (WN), wild-type insulin precursor secretion (WI), wild-type α-amylase secretion (WA), *Δhac1 *no protein secretion (dN), *Δhac1 *insulin precursor secretion (dI), *Δhac1 *α-amylase secretion (dA). Measurements are mean +/- s.e.m. (*n *= 3).

The cellular burden induced by (a) synthesizing and secreting IP and α-amylase and (b) deleting the key TF for the UPR, Hac1p, substantially affected the cells. Protein titers in WT strain were 9 mg/L and 20 mg/L, for IP and α-amylase, respectively (Figure 1a). On a per biomass basis, this is approximately half the insulin produced, and one-third the α-amylase reported for rich media [19,20]. Rich media appears to be favorable for heterologous protein production, but may present complications in downstream separations. Comparing the small and larger proteins, α-amylase was secreted in higher levels on a mass basis, but six-fold more insulin molecules were secreted (1.52 μM IP in WI compared to 0.26 μM α-amylase in WA). *Δhac1 *strains secreted significantly less protein than WT, confirming that Hac1p is important for efficient secretion (Figure 1a) [5].

Reduced specific growth rates imply impairment of cellular processes (Figure 1b). In WT yeast, IP production did not affect growth; however, α-amylase production reduced growth by 25%. This, combined with the differences in protein titers, implies that α-amylase is more challenging to fold and secrete than IP. In the *Δhac1 *background, recombinant protein strains dI and dA had approximately 20% lower growth rates compared to dN. This growth reduction occurs despite no change in specific glucose uptake rate (Additional file 1, Tables S1 and S2) pointing toward higher energy requirements to maintain homeostasis in *Δhac1 *while trying to secrete recombinant proteins. *Δhac1 *strains had overall lower final cell densities. *Δhac1 *strains produced more glycerol than WT strains implying impaired oxidative processes in the *Δhac1 *strains (Additional file 2).

### Secretory stress shifts metabolism to increase oxygen and ATP requirements

**Figure 2:**
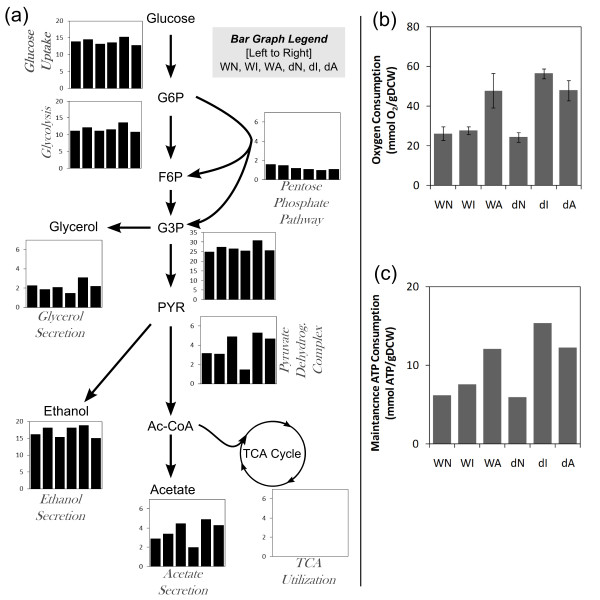
**Secretory perturbations increase oxygen and ATP consumption**. **(a) **Flux balance analysis of strains. TCA cycle is suppressed in high glucose. Bar graphs show flux in mmol/gDCW/h. Complete fluxes in Additional file 4. FBA calculations were performed using *n *= 3 measurements with errors noted in Additional file 1 (Tables **S**1 and **S**2). The average coefficient of variance for these measurements is 11%. **(b) **Oxygen required for growth. The amount of oxygen consumed for each gram of cells (DCW) formed. Oxygen measurements are mean +/- s.e.m. (*n *= 3). **(c) **Specific maintenance ATP consumption as calculated by flux balance analysis (see Materials and Methods for details of calculation). FBA calculations were performed using *n *= 3 measurements with errors noted in Additional file 1 (Table **S**1 and **S**2). The average coefficient of variance for these measurements is 11%. Wild-type, no protein secretion (WN), wild-type insulin precursor secretion (WI), wild-type α-amylase secretion (WA), *Δhac1 *no protein secretion (dN), *Δhac1 *insulin precursor secretion (dI), *Δhac1 *α-amylase secretion (dA). Gram dry cell weight (gDCW). acetyl-CoA **(Ac-CoA)**; fructose-6-phosphate **(F6P)**; glyceraldehyde-3-phosphate **(G3P)**; glucose-6-phosphate **(G6P)**; pyruvate **(PYR)**.

The physiological changes due to the secretory perturbations affect the distribution of resources through the metabolic network. The glucose uptake and range of products produced were altered by the protein production conditions (Table 1). Changes in the underlying metabolic network were estimated by flux balance analysis (FBA) using a yeast central carbon metabolism model, constrained by measured extracellular fluxes (Additional file 1, Tables S1 and S2, Additional files 3 and 4) [21]. Figure 2a shows a metabolic map of central carbon metabolism for each of the six conditions based on the exchange fluxes in Table 1 and the FBA analysis. The shift in metabolic fluxes were correlated with changes in redox requirements. As expected, the catabolic functions of the TCA cycle was predicted to have very low activity due to glucose repression [22]. Figure 2b shows that the oxygen uptake was twice as high in the strains that were growth inhibited (for example, WA, dI, dA) than those that were not. This increased oxygen uptake was not used for oxidative phosphorylation, as the biomass yields on glucose were lower in WA, dI, and dA, and it may therefore be a result of increased oxidation in connection with formation of disulfide bonds.

**Table 1:** Physiological parameters of recombinant protein secretion strains^a^

Strains^b^	Μ_max _[h^-1^]	Y_SX_	Y_SE_	Y_SG_	Y_SA_	Y_SCO2_	Carbon balance
WN	0.43 +/- 0.014	0.14 +/- 0.001	0.32 +/- 0.041	0.067 +/- 0.009	0.048 +/- 0.0005	0.30 +/- 0.019	0.89
WI	0.40 +/- 0.012	0.13 +/- 0.002	0.35 +/- 0.029	0.055 +/- 0.005	0.056 +/- 0.0047	0.30 +/- 0.014	0.92
WA	0.32 +/- 0.007	0.11 +/- 0.003	0.31 +/- 0.006	0.060 +/- 0.007	0.049 +/- 0.0023	0.30 +/- 0.002	0.84
dN	0.38 +/- 0.005	0.13 +/- 0.004	0.37 +/- 0.025	0.046 +/- 0.003	0.035 +/- 0.0046	0.29 +/- 0.020	0.91
dI	0.29 +/- 0.005	0.08 +/- 0.006	0.32 +/- 0.017	0.081 +/- 0.001	0.046 +/- 0.0011	0.31 +/- 0.007	0.84
dA	0.31 +/- 0.002	0.11 +/- 0.003	0.32 +/- 0.002	0.066 +/- 0.001	0.049 +/- 0.0009	0.30 +/- 0.004	0.85

^a^All yields (Y) are [g/g]. Glucose (S), biomass (X), ethanol (E), glycerol (G), acetate (A), carbon dioxide (CO2).

^b^Strain abbreviations as in Figure 1.

Figure 2c shows that the maintenance ATP consumption is increased in WA, dI, and dA according to FBA calculations. In WT background, WI did not consume a detectable increase in ATP, likely because IP is short and easily folded, thereby minimally taxing the translation and folding machinery. WA did increase two-fold in ATP consumption, most likely because α-amylase is 10-fold larger and likely more difficult to fold and has more disulfide bond pairing possibilities. In the *Δhac1 *background, folding efficiency is likely decreased due to ER dysfunction. With native secretion, dN did not require higher ATP maintenance consumption compared to WT. However, even the smaller, easier to fold IP resulted in ER stress that required significant ATP consumption compared to WT. dA, which was already stressed under WT, continued to show high ATP consumption. Despite the increased ATP consumption in dI and dA, little protein was secreted.

### Transcription factors controlling oxidative stress, amino acid salvaging, and expression repression are linked to secretory response

Growth phase transcriptomics measurements were carried out to identify cellular processes that were activated under the stresses of *HAC1 *deletion and recombinant protein production. *HAC1 *deletion resulted in 339 significantly changed genes in the no recombinant protein case (WN *vs*. dN). *HAC1 *deletions in the insulin strain and α-amylase strain resulted in much larger cellular responses of 1628 (WI *vs*. dI) and 1511 (WA *vs*. dA) significantly expressed genes, respectively. *KAR2 *(ER chaperone) expression was significantly reduced upon *HAC1 *deletion (↓ three-fold dN vs WTN, *P *= 1 × 10^-4^) and the four yeast protein disulfide isomerases (*PDI1, EUG1, MPD1, MPD2*) reduced an average of 2.9-fold (*P *< 0.05).

The effects of producing IP or α-amylase within a strain background (WT or *HAC1*) were not as pronounced as the effect of *HAC1 *deletion, 40 and 194 genes were significantly changed in WI (compared to WN) and WA (compared to WN). Likwsise, 74 and 90 genes were significantly changed for dI (compared to dN) and dA (compared to dN).

**Figure 3:**
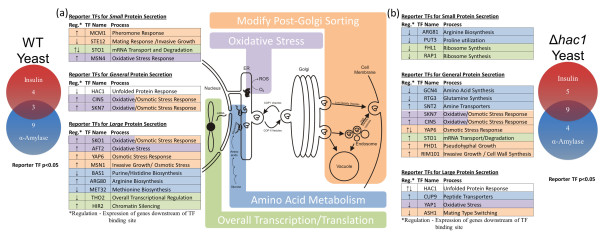
**Transcription factors activated by recombinant protein secretion**. Transcription factors were scored based on significantly changed genes in **(a) **wild-type strains, and **(b) ***Δhac1*. Venn diagram shows the number of secretory-related transcription factors activated in insulin precursor and α-amylase compared to no protein secretion. Table lists secretory-related transcription factors in small (insulin precursor) and large (α-amylase) protein secretion. Color coding indicates common secretory mechanisms as shown in diagram: modifying *trans*-Golgi network sorting (orange), oxidative stress (purple), amino acid metabolism (blue), and transcription and translation (green).

To reduce the dimensionality of the data and identify putative TFs involved in protein secretion, the Reporter Transcription Factor algorithm was used [23]. TFs were scored by the modulation in expression level of genes that the TFs bind in the upstream region according to ChIP-chip data [24]. Therefore, the score is not indicative of change in the TF expression level itself, but of the genes under its influence. Reporter TF algorithm is useful, because although the statistical significance of an individual gene may not meet an arbitrary threshold, if several genes linked to the same TF have similar behavior, the likelihood of observing the group of genes is low, making TF identification very sensitive. Figure 3 shows significant secretory process TFs shown to be involved in up- and down-regulating different cellular process under their control. Interestingly, different TFs were identified for the two different proteins. This is likely the combined effect of different protein size and number of disulfide bonds. A complete list of significant transcription factors is provided in Additional files 5 and 6.

In WT (Figure 3a), several TFs were activated by protein secretion. Oxidative and osmotic stress pathway up-regulation was common to both proteins. Oxidative stress is likely caused by ROS that is formed when Ero1p shuttles electrons to oxygen in disulfide bond formation [25]. Osmotic stress response, particular hypo-osmotic stress, strengthens the cell wall to counteract internal turgor pressure by changing the cell wall composition. This change in composition requires remodeling the secretory pathway by changing which components are trafficked to the cell wall [26]. Surprisingly, the Reporter TF algorithm found several Hac1p-influenced genes down-regulated. Genes that Hac1p binds from the ChIP-chip data that are significantly down-regulated are *KEG1, MCD4*, and *ERJ5. KEG1 *and *MCD4 *genes are involved in glycan modifications and *ERJ5 *is a secondary ER chaperone [27-29]. These genes may be influenced by other TFs not included in the ChIP-chip network. Genes known to be regulated by Hac1p (*KAR2 *and *ERO1*) were not significantly changed upon secreting recombinant protein, indicating that there is not an actual Hac1p response in the WT.

Clear differences between large and small protein secretion emerge in WT. IP stimulated modification of the TGN through *MCM1 *and *STE12*. Overall expression is reduced by altering mRNA degradation pathways via *STO1*. α-amylase had a much larger effect on the cell, as compared to IP, as was implied by physiological parameters of Figure 1 and number of altered genes. Additional oxidative and osmotic stress pathways were activated in WA, as well as a down-regulation in some amino acid synthesis pathways and overall reduction of transcription.

In the *Δhac1 *background (Figure 3b), many of the effects found in WA, have become common to both IP and α-amylase producing strains. *HAC1 *deletion clearly makes the cell more susceptible to recombinant secretion overload. Both insulin and α-amylase secretion cause considerable oxidative stress response and down-regulation of amino acid synthesis, including the general amino acid synthesis TF, Gcn4p. In dI, translational capacity repression is also employed (via Fhlp/Rap1p) and adjustments in amino acid metabolism. dA shows a mix of up- and down-regulation of genes that are controlled by Hac1p. Other TFs appear to be controlling these genes in the absence of *HAC1*. Some oxidative and osmotic stress pathways appear independent of *HAC1*. Skn7p and Cin5p were similarly activated in both WT and *Δhac1*. Oxidative and hypo-osmotic stress, while important for managing the secretory pathway, appears not to be directly managed through the UPR.

### Thermodynamic irreversibilities in redox reactions can explain increased oxidative stress in slow protein folding conditions

The increases in oxidative stress, oxygen consumption, and reduced growth observed in the study can be explained by electron transfer in ER redox pathways. Disulfide bond formation has been established to consume oxygen and produce ROS (and thereby consume cellular resources to protect against the ROS) in stoichiometric quantities with the number of disulfide bonds formed [9]. When non-native disulfide linkages are formed, these linkages must be rearranged to the correct disulfide pairings for the native protein to be folded, a process called disulfide isomerization [30].

Disulfide isomerization involves (a) breaking the non-native bond by transferring electrons to the non-native bond creating a cysteine linkage with the PDI, and (b) creating a new disulfide linkage in the nascent protein by transferring the electrons to break the PDI-nascent protein linkage. By random pairing, the native disulfide bonds are found.

Directionality in these redox reactions is determined by thermodynamic favorability through electron affinity of the potential disulfide bonds. Disulfide isomerization is redox neutral, not requiring electron donors or acceptors. However, it does require each disulfide pairing to have a lower electron affinity than the next (non-native disulfide in folding protein < PDI-folding protein disulfide < native disulfide in folding protein) to allow the electrons to transfer. Under slow folding conditions, PDI may hold the disulfide bond (oxidized state) for extended time because a native disulfide cannot be found, resulting in PDI being reduced by other moieties, likely GSH.

**Figure 4:**
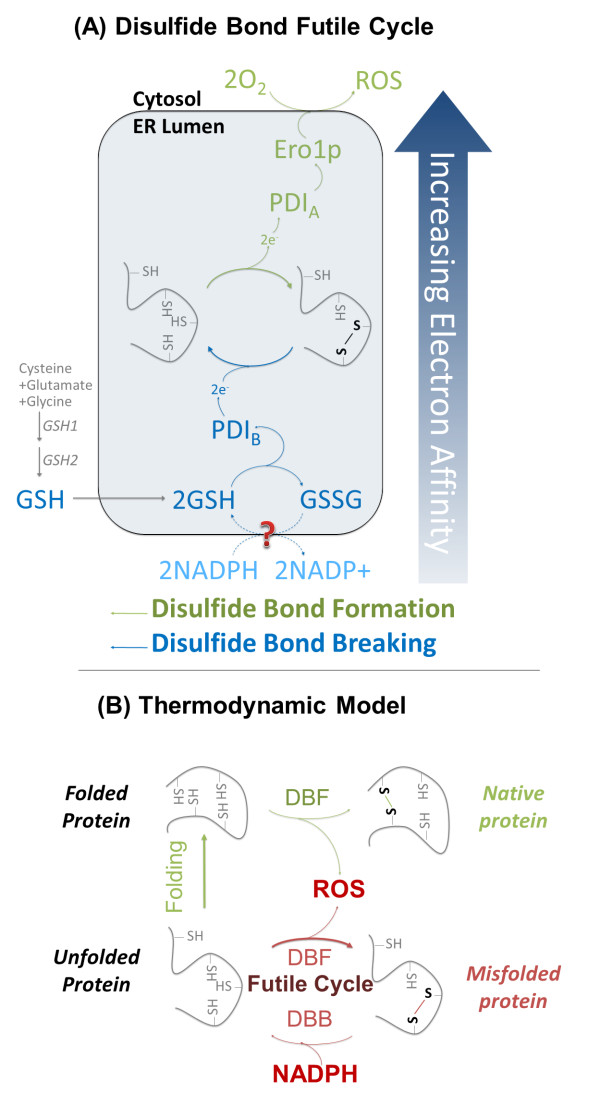
**Proposed thermodynamic model predicts non-stoichiometric reactive oxygen species produced with incorrect disulfide bond formation**. **(a) **In the model, forming and breaking an incorrect disulfide bond uses two protein disulfide isomerases (PDIs), one with electron affinity higher (PDI_A_) and one lower (PDI_B_) than the incorrect disulfide bond. In the formation phase, electrons are shuttled to molecular oxygen, resulting in ROS formation. In the breaking phase, electrons are passed from NADPH, through glutathione, to the protein. In both cases, electrons move along the electron affinity gradient. The net result is a futile cycle that is required to fix incorrect disulfide bonds, but expends redox energy. **(b) **The thermodynamic model predicts at fast folding rates near stoichiometric ROS is generated per disulfide bond formed. However, when folding rates are slow, the unfolded protein may go through many futile cycles, resulting in excess ROS. Glutathione (GSH), oxidized glutathione (GSSG), disulfide bond formation (DBF), disulfide bond breaking (DBB).

Given the observations in our experiments, and the thermodynamic reasoning immediately above, we propose a simple thermodynamic model of disulfide bond formation and breaking that explains increased oxidative stress, oxygen consumption, and reduced growth observed in our experiments. This model expands upon the mechanism by Cuozzo and Kaiser [13]. The thermodynamic model assumes there are PDI disulfide bonds that have electron affinities above and below the nascent proteins disulfide bonds (Figure 4a). The disulfide is formed by the typical oxidation pathway (Figure 4a, green) catalyzed by high electron affinity PDI (called PDI_A _here). Instead of isomerization, the incorrect disulfide is reduced by an electron donor with a low electron affinity (most likely a different PDI paralogue, called PDI_B _here) (Figure 4a, blue). The difference in electron affinity between the folding protein's cysteines and a specific PDI's cysteines can only allow the electrons to flow in one direction (toward the higher electron affinity cysteines) (Figure 4a). Therefore, a different PDI is required to form and break the incorrect disulfide bond. This futile cycle relies on a strong electron affinity gradient to complete an isomerization-like process. The net result of the futile cycle is GSH consumption and ROS production. This model implies that the ROS produced is not stoichiometrically linked to the number of disulfide bonds formed, but varies by the number of futile cycles before the correct bond is formed.

The metabolic and transcriptional data supports this model. Upon *HAC1 *deletion, ER chaperones (*KAR2*) and PDIs (*PDI1, FUG1, MPD1*, and *MPD2*) expression is reduced. This downregulation of ER chaperones and PDIs results in suppressed ER folding and disulfide bond formation in the Δ*hac1 *mutants. In the dN case, minimal oxidation stress is seen. However, when there is an increased demand for protein folding and disulfide bond formation, as is the case for dI and dA case, we see high oxygen consumption, ATP requirements, and many oxidative stress pathways being activated transcriptionally. Although both folding and disulfide bond formation is down, an imbalance toward faster disulfide bond formation compared to folding will result in futile cycles. Therefore, this disulfide/folding imbalance acts as a catalyst for drastically increasing ROS production.

Based on this thermodynamic model, the relative rates of protein folding and disulfide bond formation for nascent peptides have important consequences for oxidative stress (Figure 4b). When folding is faster than disulfide bond formation, ROS is produced in near one-to-one amounts with the disulfide bonds formed. Under these conditions, isomerization may be more efficient to resort incorrect disulfide bonds, as native structures with low electron affinity disulfide pairs are favored, and isomerization does not produce ROS. However, when folding is slow compared to disulfide bond formation, as is the case when the protein folding machinery gets overloaded, the nascent peptides cycles through the futile redox cycle producing ROS and consuming GSH in excess to the final number of disulfide bonds formed. The physiological result of a high disulfide bond formation to ER folding rate is oxidative damage to a broad range of cellular proteins and consumption of reducing equivalents that could otherwise be used for anabolism.

## Discussion

In this study, we have identified biological mechanisms related to protein synthesis and secretion by introducing perturbations to the cell, in the form of *HAC1 *deletion and different recombinant protein expression, and measuring the system level cellular responses, via transcriptomics and metabolic fluxes. These measurements, combined with data analysis algorithms, Reporter TF algorithm and FBA, were able to identify cellular adjustments in (a) overall expression level, (b) post-Golgi sorting, (c) amino acid biosynthesis and savaging, and (d) oxidative stress. These biological effects are a result of the combined influence of protein synthesis and trafficking through the secretory pathway.

Overall transcription and translation were repressed in response to α-amylase expression (a larger protein) and in the *Δhac1 *strains with any recombinant protein secretion. Repressing overall expression is a broad spectrum response used to adjust the rates of all other cellular processes to match the reduced folding capacity in the ER. Several mechanisms were used to alter overall expression: repressing mRNA synthesis, increasing mRNA degradation rates, and repressing protein translation rates through reducing ribosome numbers. Specifically, mRNA concentrations are lowered by decreasing RNA polymerase accessibility (*HIR2*), inhibiting transcriptional elongation (*THO2*), and controlling RNA degradation (*STO1*) [31,32]. Ribosome concentration, and thereby translation rates, can be reduced by the TFs Fhl1p and Rap1p which control expression of rRNA and ribosomal proteins [33]. This is seen in IP production in Δ*hac1 *strain, both by the reporter TFs (Figure 3) and by expression of ribosomal proteins (Additional file 7). In this context, extrachromosomal plasmids offer advantages over chromosomal expression. *HIR2*, whose mechanism is to silence the chromosome, would not affect extrachromosomal plasmids. Increased recombinant protein secretion would be accomplished by silencing native ER genes, while recombinant, plasmid-born gene would not be affected.

Pronounced adjustments to the TGN were observed in the transcriptome in all conditions. TFs involved in pheromone responses (*STE12, MCM1, ASH1*), invasive/pseudohyphal growth (*STE12, MSN1, PHD1, RIM101*), and osmotic stress (*CIN5, SKN7, SKO1, YAP6, MSN1*) were all identified by the Reporter TF algorithm and point to an underlying set of activities that are required to increase the traffic of secretory vesicles to the membrane. Invasive, pseudohyphal, and filamentous growth morphologies have a high surface to volume ratio and inherently require higher Golgi-to-cell membrane trafficking rates to supply cell membrane and cell wall components for growth. These altered morphologies can be activated through the filamentous and invasive response elements (FREs) [34] bound by *STE12 *and used to regulate *PHD1 *[35]. *HAC1 *deletion has been shown to cause filamentous growth [36].

Osmotic stress TFs are also responsible for affecting protein secretion, as the external cell wall must be strengthened in response to hypo-osmotic conditions, thereby requiring an efficient secretory pathway to ferry cell wall proteins [26]. *MSN1 *is known to induce starch degradation, requiring the actions necessary to secrete the appropriate enzymes through filamentous growth activation [37]. *SKN7 *has a dual role in invasive growth and osmotic stress [38]. Although osmotic stress TFs are commonly associated with the hyper-osmotic glycerol (HOG) pathway, Ypd1p can phosphorylate Skn7p, signaling the hypo-osmotic stress pathway [39]. Because there were no apparent hypo-osmotic conditions in this study, this indicates that these TFs are not directly controlled by osmotic conditions, but possibly through a secondary response to upregulation and increased secretion of cell wall proteins.

TGN TFs and/or the genes they regulate are possible targets for increasing Golgi-to-cell membrane trafficking. In *S. cerevisiae*, recombinant protein intended for secretion has been found mis-trafficked to the vacuole. This has been shown for insulin and green fluorescent protein secretion in yeast [40,41]. Proteins involved in vesicle trafficking, namely Sly1 and Munc18 have been found to increase recombinant secretory rates in Chinese hamster ovarian (CHO) and several mammalian cell lines [42,43]. It is likely that similar proteins are present in yeast and could be exploited for improving protein secretion.

Significant alterations in amino acid metabolism were observed, particularly in the *Δhac1 *strains. *De novo *amino acid synthesis (*GCN4, BAS1, MET32, ARG81, RTG3*) was suppressed. On the surface, this appears contradictory, as increased amino acid requirements should be observed with recombinant protein production. However, this decrease in *de novo *amino acid synthesis is accompanied by observed increases in scavenging mechanisms for amino acids (*SNT2, CUP9, PUT3*). High scavenging rates and decrease synthesis imply high protein degradation rates where the degraded proteins result in available amino acids for scavenging; reducing the need for newly synthesized amino acids. This is consistent with either ERAD, a process where proteins that are stalled in the ER are transported back into the cytoplasm for degradation by the proteosome, or vacuolar-localized protein degradation. In either case, the cell is expending energy on synthesizing proteins that are ultimately degraded. These effects appear in the strains that are the slowest growing with the highest ATP requirements (Figures 1b and 2b). In these cases the ER folding capacity is likely saturated, resulting in ER holdup and ERAD.

Oxidative stress TFs were also found in all conditions. Several were dual oxidative/osmotic stress TFs (*CIN5, SKN7, SKO1*), and others were dedicated to oxidative stress only (*AFT2, YAP1*). TFs were found in all three of the major oxidative stress signaling pathways, (a) the Hog1 MAPK pathway (where *SKO1 *is the DNA binding agent), (b) Sln1 pathway (where *SKN7 *is the DNA binding agent), and (c) *YAP1 *and *CIN5*, which directly sense oxidative stress and bind DNA [44]. The cell's control machinery appears to have hard-wired oxidative stress responses to increased secretory demand, as oxidative/hypo-osmotic pathways have a high degree of overlap, which is appropriate because increased secretion of cell wall proteins will result in higher oxidative stress. In particular, Skn7p, which has already been mentioned for its role in managing secretory pathway directly in an osmotic stress pathway, can also activate oxidative stress response genes [45].

Oxidative stress was pronounced with all secretory perturbations and has been identified in other studies to be associated with secretory stress [1,17]. Futile cycling may be the dominant disulfide resorting pathway when folding is limited. In previous studies, oxidative stress, induced by tunicamycin, a N-linked glycosylation inhibitor, increased with ER stress, despite no increase in the net disulfide bond formation demand [17]. The futile cycle does predict non-stoichiometric ROS formation, while isomerization does not. ROS can be formed at potentially limitless amounts through multiple rounds of disulfide formation and breaking. This will occur under conditions where the rate of folding is slow, a result of proteins that are specifically difficult to fold, or a result of the overall ER folding capacity being saturated. As well, futile cycling will increase as the number of available cysteine residues available for disulfide bonding increase, as is the case for α-amylase, due to the extended amount of isomerization that may be needed to form the correct disulfide bonds.

One implication of the proposed thermodynamic model is that PDI paralogues, or cysteines within a PDI, must exist at different electron affinities that are above and below the electron affinity of the protein to be folded. Although *in vivo *redox potentials of PDI cysteine pairs were not measured, from first principles it would appear highly likely that these PDIs would need different redox potentials to carry out isomerization. In Figure 4a, we assume that only PDIs interact with the folding protein. This appears the case, as kinetic rates for direct glutathione oxidation/reduction are too slow to be physiologically relevant [9]. Electron affinity (and therefore redox potential) is broadly determined by the proximity of the two cysteines, with the proximity determined by the current structure of the protein [46]. Cysteines that are in the correct orientation will have a low electron affinity and easily form disulfide bonds, while cysteines that are not in the correct orientation will have a high electron affinity and will have unstable disulfide bonds. Therefore, the electron affinity of a correctly folded/correct disulfide bond would be lower than that of a misfolded or incorrect disulfide bond. This difference in electron affinity may allow PDIs to selectively break disulfides with high electron affinity (incorrect bonds), but not disulfide bonds with low affinity (correct bonds).

The need for different PDIs to form or break disulfide bonds may explain the need for many PDI homologues in the ER, each with different structures, and therefore different electron affinities. These PDIs can only span a finite range of electron affinities, and there may be implications for proteins that have disulfide pairs with electron affinities higher than the highest PDI or lower than the lowest PDI. If no PDI has a lower electron affinity than an incorrect disulfide bond, then the disulfide bond cannot be broken and the protein is terminally misfolded. As well, a protein that has a native disulfide pairing with an electron affinity higher than any PDI cannot form a bond. This may be the case when recombinant proteins are being processed in the ER.

Futile cycling as a large potential ROS source has broad implications on the cell. Tu and Weissman predict Ero1p-produced ROS that is one-to-one with disulfide bond formation could attribute approximately 25% of cellular ROS to the secretory pathway [1]. Therefore, even larger ROS production is likely if the futile cycle is the dominant disulfide resorting pathway under folding stress. This also has implications on GSH and possibly NADPH availability, as it is doubly consumed (a) by the reduction of ROS and (b) directly in the futile cycle. The futile cycle limits reducing equivalents needed for anabolic processes, and may explain the reduced growth rates observed in folding stressed strains (WA, dI, and dA).

In all, Figure 4b highlights that the relative rates of two processes, protein folding and disulfide bond formation, must be kept in balance to avoid significant cellular stress. If disulfide bond formation is fast compared to folding, high futile cycle use will result in high ROS formation, NADPH loss, and high protein degradation as a result of ERAD. This scenario is observed in the *Δhac1 *strains dI and dA.

The engineering implications for protein secretion become much clearer with this understanding of protein folding to disulfide bond formation ratio. When overexpressing a recombinant protein, an optimal expression must be found, where transcription is as high as possible without overloading the ER folding capacity and sending the cell into an oxidative stressed state. This optimal expression level will be different for different proteins, as protein folding rates will vary according to the protein size and structure. We see this in comparing IP and α-amylase expression. The concept of an optimal expression has been identified heuristically, in the present study we identify the competing molecular effects that could define these phenomena [47]. This optimal expression ratio extends to recombinant proteins that do not have disulfide bonds. For recombinant proteins without disulfide bonds, recombinant protein folding in the ER will consume folding resources, thus slowing down folding rates. Although the recombinant protein has no disulfide bonds, many native proteins still require disulfide bonds. Because of this, the folding to disulfide bond formation ratio will be disturbed, resulting in similar ROS stress.

To maintain an optimal ratio, either protein folding rates must increase or oxidation rates decrease. Overexpression of chaperones that increase folding capacity has successfully been used to increase protein secretion [6,48]. For particularly large or difficult to fold proteins this may not be adequate. A new approach may be to limit the oxidation rate of Ero1p to slow down the first step of the futile cycle. This would be done in concert with repressing ERAD, as proteins would have long retention times in the ER. In this scenario, recombinant proteins would be slowly folded, albeit without high cellular stresses. This would result in longer overall process times, but may be required for difficult to fold proteins.

## Conclusion

In this study, we identified post-Golgi vesicle sorting, high protein degradation rates, repressed overall expression, and oxidative stress in response to +/- UPR strains secreting different sized recombinant protein. These processes were identified through scoring TFs and estimating alteration to the metabolic network. These observations imply our proposed futile cycling is the dominant disulfide resorting pathway in the ER and explains non-stoichiometric ROS formation seen in our study and elsewhere. The futile cycle model, producing ROS and consuming GSH, has a clear thermodynamic driving force compared to disulfide bond isomerization. If correct, futile cycling is likely the dominant mechanism under secretory stress. This interplay between protein folding and futile cycling sheds light on a largely empirical understanding of engineering protein secretion and implies the relative rates of protein folding and disulfide bond formation are critical to maintaining cellular homeostasis. This increased molecular understanding of the secretory pathway should allow for more insightful design of secretory engineering strategies.

## Methods

### Strains and media

All experiments were performed in the background of CEN.PK 113-5D (*MAT ***a ***SUC2 MAL2*-*8*^c ^*ura3*-*52*, P. Kötter, Frankfurt, Germany) [49]. Genomic DNA from Y05650 (BY4741; Mat a; *his*3D1; *leu*2D0; *met*15D0; *ura*3D0; YFL031w::kanMX4, obtained from EUROSCARF) was used as a template for the *HAC1 *knockout cassette. Standard molecular biology techniques were used [50] and all plasmids were maintained in *Escherichia coli *DH5α in Luria Bertani (LB) broth with 80 mg/L ampicillin. PCR primers are listed in Additional file 8.

### Cloning

Genomic DNA was purified from Y05650 using Fast DNA Spin Kit for Soil (MP Biomedicals Solon, OH, USA). A 2.6 kb DNA fragment containing the genomic replacement of *HAC1 *with KanMX and flanking regions was amplified by PCR using primers KT007/KT008 (Additional file 8). The *HAC1*::kanMX4 fragment was integrated at the *HAC1 *loci of CEN.PK 113-5D by standard yeast transformation [51] and selected on 200 mg/L G418 to create the *Δhac1 *strain. Correct integration was confirmed by PCR.

DNA coding for an insulin precursor with a Yap3 pre-leader sequence and the TA57 pro-leader sequence and spacers as described [18] for correct secretory processing was synthesized with optimal codon usage for yeast and delivered on plasmid pUC57-Yap3Insulin (GenScript Co. Piscataway, NJ, USA) (Additional file 9 for sequence). α-Amylase DNA was amplified from *Saccharomyces kluyveri *YKM37 [52] using LZH018 and LZH039. The pre-pro-leader was amplified from pUC57-Yap3Insulin using primers LZH015 and LZH016. The pre-pro-leader was connected to the α-amylase by fusion PCR of the two segments together using primers LZH015 and LZH039 [53]. The pre-pro-insulin and pre-pro-amylase were cloned into the SpeI/SalI or SpeI/EcoRI sites of p426GPD, respectively, downstream of the constitutive GAPDH promoter [54], to create pYapIns and pYapAmy. Plasmids p426GPD, pYapIns, and pYapAmy were transformed into CEN.PK 113-5D and *Δhac1 *strains by standard methods [51].

### Fermentor conditions

Strains were grown in SD-2xSCAA [55], containing 20 g/L glucose, 6.7 g/L yeast nitrogen base minus amino acids (Formedium, Norfolk, UK), 2 g/L KH_2_PO_4 _(pH = 6 by NaOH), 190 mg/L Arg, 108 mg/L Met, 52 mg/L Tyr, 290 mg/L Ile, 440 mg/L Lys, 200 mg/L Phe, 1260 mg/L Glu, 400 mg/L Asp, 380 mg/L Val, 220 mg/L Thr, 130 mg/L Gly, 400 mg/L Leu, 40 mg/L Trp, 140 mg/L His, 1 g/L bovine serum albumin. Five hundred mL of medium was inoculated in a 1 L bioreactor (DasGip, Jülich, Germany) at 30°C, 600 rpm agitation, 30 standard L/h air flow, pH controlled at 6 by KOH (2 M). Strains were inoculated to an A_600 _= 0.01 from late exponential phase cultures and A_600 _was measured throughout the cultivation. Dry cell weight (DCW) was measured by filtering 5 mL of culture broth through a 0.45 μm nitrocellulose filter and measuring the increased weight of the dry filter. Glucose, ethanol, glycerol, and acetate were measured using a Summit HPLC (Dionex, Thermo Scientific, Waltham, MA, USA) with an Aminex HPX-87H column (Bio-Rad, Hercules, CA, USA). Carbon dioxide and oxygen levels were measured in the off-gas and dissolved oxygen was monitored. Transcriptome samples were taken after 5+ doublings at A_600 _= 1.0-1.4. Triplicate fermentations were carried out for each strain.

### Protein quantification

Insulin was measured by a modification of the assay by Snell *et al. *[56]. One mL of cell culture was centrifuged at 4000 × g for 4 min. Eight parts supernatant was added to one part 0.1 N HCl and 5.5 μM sodium azide and stored at 4°C until measurement. Insulin concentration was determined by HPLC using a Luna 5 μ C18(2) (250 mm × 4.6 mm) (Phenomenex, Torrance, CA, USA) column and gradient-based elution. Buffer A contained 68 mM phosphoric acid, 0.2 M sodiumsulphate and 10% (w/v) acetonitrile in water, and Buffer B contained 50% acetonitrile in water. HPLC was run with 25 μL injections at 1 mL/min and 50°C. Gradient protocol: 20% B for 10 min. Linear gradient from 20% B to 60% B over 10 min. Hold at 60% B for 5 min and then to 20% B for 3 min to re-equilibrate for next sample. Insulin standards eluted at 22.6 min and insulin precursor at 20.0 min. HPLC peaks were verified to be the correct protein by SDS-PAGE. Human insulin was used as a standard (Sigma, St. Louis, MO, USA).

α-amylase concentration was calculated from enzyme activity. α-amylase activity was measured using the Ceralpha kit (Megazyme K-CERA, Wicklow, Ireland) using α-amylase from *Aspergillus oryzae *(Sigma, St. Louis, MO, USA) as a standard. This conversion was calculated using a 1.79 U/mg (weight includes salts and purified protein) standard from Sigma using the Protein 80 chip on the Bioanalyzer (Agilent, Santa Clara, CA, USA). By this, α-amylase was found to be 0.0257 g α-amylase/g total.α-amylase activity was then converted to mass using 70 U/mg α-amylase protein.

### Transcriptome analysis

Samples for microarray were taken as described previously and stored at -80°C until processing [57]. RNA was isolated using the RNeasy Minikit (Qiagen, Valencia, CA, USA). Cells were lysed in RNeasy RLT buffer using Lysing Matrix C (MP Biomedicals Solon, OH, USA) in a Fast Prep 24 (MP Biomedicals Solon, OH, USA) as follows: 20 s at speed 6, 1 min at 4°C, 20 s at speed 6. RNA was processed to aRNA using the Genechip 3' IVT Express Kit (Affymetrix, Santa Clara, CA, USA) and hybridized/scanned on the Yeast Genome 2.0 Array (Affymetrix, Santa Clara, CA, USA) following commercial protocols to create CEL files.

Images were analyzed using R 2.10.1 statistical software and the 'affy' and 'limma' packages as described previously [58]. Briefly, background normalization was carried out using robust multi-array (RMA) average method with perfect match (PM) probes only. Interchip normalization used the qspline algorithm with median polish summary method. Statistical analysis was carried out by comparison of triplicate bioreactor measurements for each strain. Emperical Bayesian statistics were used to moderate standard errors within each gene and Benjamini-Hochberg's method to adjust for multiple testing. Microarray data was submitted to the GEO database and have accession number GSE27062 (see http://www.ncbi.nlm.nih.gov/geo/query/acc.cgi?token=dpyzfywysoqecbk&acc=GSE27062).

### Reporter transcription factor analysis

Transcription factor activity was scored using the Reporter Effector algorithm [23]. Transcription factor-DNA interactions were gathered from ChIP-chip with *P *< 0.001 [24]. Significant interactions were found for 176 transcription factors regulating 3,796 genes for a total of 10,849 unique interactions. Gene *P *values from comparing different strains were used to score transcription factors that were known to bind to the upstream DNA. Transcription factors with *P *< 0.05 of being activated between conditions are reported.

### Flux balance analysis

Estimates of intracellular reaction rates were performed using measured exchange fluxes of glucose, ethanol, acetate, glycerol, and carbon dioxide. Model-based error correction was used to close carbon and electron balances [59]. Flux balance analysis was carried out using a 85 reaction model of yeast central carbon metabolism and biomass yield were used [21]. Additional file 4 contains the complete results of the analysis which are used to estimate ATP consumption in the different strains.

## Abbreviations

ATP: adenosine triphosphate; dA: *Δhac1 *secreting α-amylase; dI: *Δhac1 *secreting IP; Dn: *Δhac1 *with empty vector; DNA: deoxyribonucleic acids; ER: endoplasmic reticulum; ERAD: ER-associated degradation; FBA: flux balance analysis; FRE: filamentous and invasive responsive elements; GSH: reduced glutatathione; HOG: hyper-osmotic glycerol; IP: insulin precursor; mRNA: messenger RNA; NADPH: nicotinamide adenine dinucleotide phosphate; PDI: protein disulfide isomerase; RNA: ribonucleic acid; ROS: reactive oxygen species; rRNA: ribosomal RNA; TF: transcription factor; TGN: trans-Golgi network; UPR: unfolded protein response; WA: WT secreting α-amylase; WI: WT secreting IP; WN: WT with empty vector; WT: wild-type.

## Competing interests

The authors declare that they have no competing interests.

## Authors' contributions

KT, DP, and JN designed the experiment. KT and ZL carried out all cloning, fermentations, and analytical measurements. KT did primary calculations in transcriptomics and metabolic flux data. KT, ZL, DP, and JN analyzed data and wrote the manuscript. DP and JN supervised the research. All authors have read and approved of the final manuscript.

## Supplementary Material

Additional file 1 **Measured exchange fluxes in strains**. Measured metabolite exchange fluxes for strains used in this study. Click here for file

Additional file 2 **Final glycerol concentration of WT and *Δhac1 *strains**. Measured glycerol titers at end of fermentation for strains used in this study. Click here for file

Additional file 3 **Estimated exchange fluxes**. Metabolite exchange fluxes as estimated by error-correction algorithm for strains in this study. Click here for file

Additional file 4 **Intracellular fluxes for metabolic network**. Flux balance analysis estimates of internal fluxes for strains in thus study. Click here for file

Additional file 5 **Reporter TFs for WT protein secretion**. Transcription factors activated by recombinant protein secretion in wild-type background. Click here for file

Additional file 6 **Reporter TFs for Δ*hac1 *protein secretion**. Transcription factors activated by recombinant protein secretion in Δ*hac1 *background. Click here for file

Additional file 7 **Expression profiles for ribosomal proteins**. mRNA concentrations for yeast ribosomal proteins as determined by DNA microarray. Click here for file

Additional file 8 **Oligonucleotides used in this study**. PCR primers used for cloning and validation. Click here for file

Additional file 9 **Synthesized insulin precursor DNA sequence**. DNA sequence for insulin precursor used in this study. Click here for file
