# Supplementary material for: Imbalance of heterologous protein folding and disulfide bond formation rates yields runaway oxidative stress
Source: BMC Biol. 2012 Mar 1;10:16. doi: 10.1186/1741-7007-10-16 (PMC3310788; doi:10.1186/1741-7007-10-16)
Supplement: Additional file 1 — Measured exchange fluxes in strains. Measured metabolite exchange fluxes for strains used in this study. [file 1741-7007-10-16-S1.DOC]

## Additional File 1 - Measured Exchange Fluxes in Strains

| **Table S1 – Measured Exchange Fluxes in Wild Type Strains*** | | | | | | | | | |  |
| --- | --- | --- | --- | --- | --- | --- | --- | --- | --- | --- |
|  | **WN** | | **WI** | | | **WA** | | | |  |
| **Biomass Formation**** | 16.8 | +/- | 0.97 | 15.4 | +/- | 0.79 | 12.5 | +/- | 0.50 |  |
| **Glucose Uptake** | 16.8 | +/- | 0.81 | 17.1 | +/- | 1.29 | 16.8 | +/- | 0.36 |  |
| **O_2_ Uptake** | 11.2 | +/- | 2.31 | 10.9 | +/- | 0.73 | 15.6 | +/- | 5.05 |  |
| **Ethanol Secretion** | 20.6 | +/- | 3.94 | 23.2 | +/- | 2.18 | 20.3 | +/- | 1.11 |  |
| **Glycerol Secretion** | 2.2 | +/- | 0.58 | 1.8 | +/- | 0.43 | 2.0 | +/- | 0.42 |  |
| **Acetate Secretion** | 2.4 | +/- | 0.12 | 2.8 | +/- | 0.28 | 2.5 | +/- | 0.23 |  |
| **CO_2_ Secretion** | 19.2 | +/- | 0.06 | 21.0 | +/- | 0.35 | 20.4 | +/- | 0.06 |  |

| **Table S2 - Measured Exchange Fluxes in** *Δhac1* **Strains*** | | | | | | | | | |  |
| --- | --- | --- | --- | --- | --- | --- | --- | --- | --- | --- |
|  | **dN** | | **dI** | | | **dA** | | | |  |
| **Biomass Formation**** | 14.9 | +/- | 0.32 | 11.1 | +/- | 0.33 | 11.9 | +/- | 0.14 |  |
| **Glucose Uptake** | 16.2 | +/- | 1.26 | 19.2 | +/- | 1.92 | 16.1 | +/- | 0.91 |  |
| **O_2_ Uptake** | 9.8 | +/- | 2.07 | 16.1 | +/- | 0.72 | 14.7 | +/- | 2.88 |  |
| **Ethanol Secretion** | 23.5 | +/- | 4.41 | 24.1 | +/- | 4.67 | 20.0 | +/- | 1.10 |  |
| **Glycerol Secretion** | 1.5 | +/- | 0.15 | 3.0 | +/- | 0.27 | 2.1 | +/- | 0.11 |  |
| **Acetate Secretion** | 1.7 | +/- | 0.52 | 2.7 | +/- | 0.26 | 2.3 | +/- | 0.09 |  |
| **CO_2_ Secretion** | 19.3 | +/- | 3.71 | 24.1 | +/- | 3.00 | 19.9 | +/- | 0.79 |  |

*Fluxes reported as mmol/gDCW/h

**Biomass molecular formula – CH_1.94_O_0.52_N_0.25_
