# Supplementary material for: Imbalance of heterologous protein folding and disulfide bond formation rates yields runaway oxidative stress
Source: BMC Biol. 2012 Mar 1;10:16. doi: 10.1186/1741-7007-10-16 (PMC3310788; doi:10.1186/1741-7007-10-16)
Supplement: Additional file 2 — Final glycerol concentration of WT and Δhac1 strains. Measured glycerol titers at end of fermentation for strains used in this study. [file 1741-7007-10-16-S2.DOC]

## Additional File 2. – Final Glycerol Concentration of WT and *Δhac1* strains


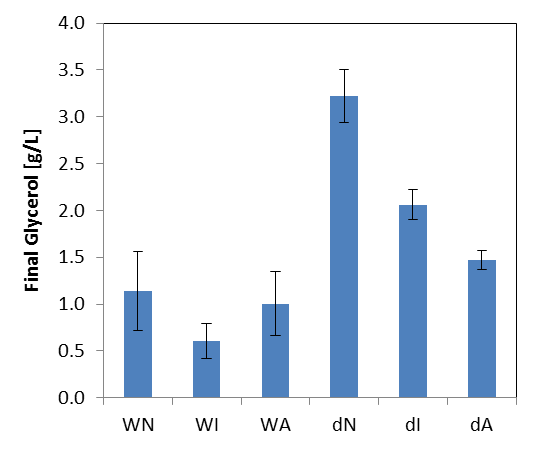


Glycerol concentration at end of fermentation. *Δhac1* strains accumulated more glycerol than WT strains. Recombinant protein production reduced glycerol accumulation in both *Δhac1* and WT.
