# Supplementary material for: Imbalance of heterologous protein folding and disulfide bond formation rates yields runaway oxidative stress
Source: BMC Biol. 2012 Mar 1;10:16. doi: 10.1186/1741-7007-10-16 (PMC3310788; doi:10.1186/1741-7007-10-16)
Supplement: Additional file 3 — Estimated exchange fluxes. Metabolite exchange fluxes as estimated by error-correction algorithm for strains in this study. [file 1741-7007-10-16-S3.DOC]

| **Additional File 3 – Estimated^†^ Exchange Fluxes in Strains*** | | | | | | | |
| --- | --- | --- | --- | --- | --- | --- | --- |
|  | **WN** | **WI** | **WA** | **dN** | **dI** | **dA** |  |
| **Biomass Formation**** | 16.9 | 15.5 | 12.6 | 15.0 | 11.2 | 12.0 |  |
| **Glucose Uptake** | 13.9 | 14.6 | 13.2 | 13.6 | 15.3 | 12.8 |  |
| **O_2_ Uptake** | 4.3 | 4.1 | 7.2 | 2.7 | 7.2 | 6.7 |  |
| **Ethanol Secretion** | 16.2 | 18.3 | 15.5 | 18.2 | 18.9 | 15.1 |  |
| **Glycerol Secretion** | 2.3 | 1.9 | 2.1 | 1.5 | 3.1 | 2.2 |  |
| **Acetate Secretion** | 2.5 | 2.9 | 2.5 | 1.8 | 2.7 | 2.4 |  |
| **CO_2_ Secretion** | 22.3 | 23.9 | 24.1 | 22.3 | 28.0 | 23.4 |  |

*Fluxes reported as mmol/gDCW/h

**Biomass molecular formula – CH_1.94_O_0.52_N_0.25_

^†^Flux corrected by macroscopic elemental balances (Wang and Stephanopoulos, 1983). This is required to ensure feasible flux balance analysis (FBA) solutions can be found.
