# Supplementary material for: Imbalance of heterologous protein folding and disulfide bond formation rates yields runaway oxidative stress
Source: BMC Biol. 2012 Mar 1;10:16. doi: 10.1186/1741-7007-10-16 (PMC3310788; doi:10.1186/1741-7007-10-16)
Supplement: Additional file 4 — Intracellular fluxes for metabolic network. Flux balance analysis estimates of internal fluxes for strains in thus study. [file 1741-7007-10-16-S4.DOC]

| **Additional File 4 – Intracellular fluxes for metabolic network *** | | | | | | |
| --- | --- | --- | --- | --- | --- | --- |
| **Reaction Name^†^** | **WTN** | **WTI** | **WTA** | **dN** | **dI** | **dA** |
| **'HTX-GAL2'** | 0.0139 | 0.0146 | 0.0132 | 0.0136 | 0.0153 | 0.0128 |
| **'PFK1-2'** | 0.0153 | 0.0166 | 0.0173 | 0.0149 | 0.0203 | 0.0167 |
| **'FBP1'** | 0.0031 | 0.0035 | 0.0054 | 0.0027 | 0.0061 | 0.0051 |
| **'PCK1'** | 0.0030 | 0.0034 | 0.0049 | 0.0025 | 0.0056 | 0.0047 |
| **'CIT2'** | 0.0007 | 0.0005 | 0.0017 | 0.0004 | 0.0018 | 0.0016 |
| **'ALD3'** | 0.0008 | 0.0008 | 0.0007 | 0.0014 | 0.0006 | 0.0006 |
| **'ALD6'** | 0.0005 | 0.0006 | 0.0008 | 0.0004 | 0.0009 | 0.0008 |
| **'ALD5_m'** | 0.0004 | 0.0003 | 0.0003 | 0.0005 | 0.0002 | 0.0003 |
| **'CIT1-3_m'** | 0.0000 | 0.0000 | 0.0000 | 0.0000 | 0.0000 | 0.0000 |
| **'IDH1-2_m'** | 0.0000 | 0.0000 | 0.0000 | 0.0000 | 0.0000 | 0.0000 |
| **'IDP1_m'** | 0.0000 | 0.0000 | 0.0000 | 0.0000 | 0.0000 | 0.0000 |
| **'MAE1_m'** | 0.0005 | 0.0005 | 0.0004 | 0.0003 | 0.0004 | 0.0004 |
| **'GPD1-2'** | 0.0084 | 0.0078 | 0.0116 | 0.0054 | 0.0123 | 0.0111 |
| **'PYK2-CDC19'** | 0.0250 | 0.0274 | 0.0266 | 0.0254 | 0.0308 | 0.0257 |
| **'SOL1-2-3-4'** | 0.0016 | 0.0015 | 0.0012 | 0.0011 | 0.0010 | 0.0011 |
| **'GND1-2'** | 0.0016 | 0.0015 | 0.0012 | 0.0011 | 0.0010 | 0.0011 |
| **'GUT1'** | 0.0039 | 0.0047 | 0.0065 | 0.0037 | 0.0070 | 0.0061 |
| **'GUT2'** | 0.0061 | 0.0059 | 0.0095 | 0.0038 | 0.0092 | 0.0089 |
| **'RevGUT'** | 0.0061 | 0.0066 | 0.0085 | 0.0052 | 0.0101 | 0.0082 |
| **'IDP2-3'** | 0.0004 | 0.0004 | 0.0003 | 0.0004 | 0.0003 | 0.0003 |
| **'ICL1-2'** | 0.0003 | 0.0001 | 0.0014 | 0.0000 | 0.0015 | 0.0013 |
| **'MLS1-2'** | 0.0003 | 0.0001 | 0.0014 | 0.0000 | 0.0015 | 0.0013 |
| **'MDH2-3'** | 245.9088 | 259.2843 | 265.4224 | 312.4964 | 300.7742 | 264.2435 |
| **'PDC1-5-6'** | 0.0175 | 0.0196 | 0.0169 | 0.0200 | 0.0204 | 0.0165 |
| **'ACH1'** | 0.0029 | 0.0034 | 0.0045 | 0.0020 | 0.0049 | 0.0043 |
| **'ACS1-2'** | 0.0018 | 0.0019 | 0.0035 | 0.0020 | 0.0038 | 0.0033 |
| **'PDA1-PDB1_m'** | 0.0032 | 0.0031 | 0.0049 | 0.0015 | 0.0053 | 0.0047 |
| **'KGD1_m'** | 0.0000 | 0.0000 | 0.0000 | 0.0000 | 0.0000 | 0.0000 |
| **'M_Suc-FumSFC1'** | 197.4934 | 208.0281 | 215.0363 | 247.6529 | 251.9829 | 211.5655 |
| **'M_ATPACC1-3-PET9'** | 0.0093 | 0.0086 | 0.0160 | 0.0055 | 0.0156 | 0.0150 |
| **'Oxid2'** | 0.0013 | 0.0014 | 0.0020 | 0.0010 | 0.0022 | 0.0019 |
| **'Oxid3'** | 0.0009 | 0.0009 | 0.0014 | 0.0007 | 0.0015 | 0.0013 |
| **'Oxid4'** | 0.0014 | 0.0013 | 0.0021 | 0.0009 | 0.0021 | 0.0020 |
| **'Oxid5'** | 0.0050 | 0.0046 | 0.0089 | 0.0028 | 0.0085 | 0.0083 |
| **'Glycerol_transp'** | 0.0023 | 0.0019 | 0.0021 | 0.0015 | 0.0031 | 0.0022 |
| **'Acetate_transport'** | 0.0025 | 0.0029 | 0.0025 | 0.0018 | 0.0027 | 0.0024 |
| **'Ethanol_transport'** | 0.0162 | 0.0182 | 0.0154 | 0.0182 | 0.0189 | 0.0151 |
| **'CO2_T'** | 0.0222 | 0.0239 | 0.0241 | 0.0223 | 0.0280 | 0.0233 |
| **'Maintenance'** | 0.0036 | 0.0040 | 0.0053 | 0.0031 | 0.0061 | 0.0051 |
| **Additional File 6 – Intracellular fluxes for metabolic network (cont’d)*** | | | | | | |
| **Reaction Name^†^** | **WTN** | **WTI** | **WTA** | **dN** | **dI** | **dA** |
| **'Vgrowth'** | 0.0000 | 0.0000 | 0.0000 | 0.0000 | 0.0000 | 0.0000 |
| **'M_Eth'** | 245.9080 | 259.2839 | 265.4188 | 312.4971 | 300.7699 | 264.2400 |
| **'M_pyruvate'** | 0.0027 | 0.0026 | 0.0045 | 0.0011 | 0.0049 | 0.0043 |
| **'M_Mal-CitCTP1'** | 0.0000 | 0.0000 | 0.0000 | 0.0000 | 0.0000 | 0.0000 |
| **'M_MalDIC1'** | 498.3602 | 539.3023 | 553.8583 | 645.2625 | 622.0061 | 550.6265 |
| **'M_OxOAC1'** | 245.9088 | 259.2847 | 265.4201 | 312.4967 | 300.7715 | 264.2413 |
| **'M_SucDIC1'** | 252.4520 | 280.0182 | 288.4386 | 332.7662 | 321.2351 | 286.3856 |
| **'Pyruvate_transport'** | 0.0000 | 0.0000 | 0.0000 | 0.0000 | 0.0000 | 0.0000 |
| **'Succinate_transport'** | 0.0000 | 0.0000 | 0.0000 | 0.0000 | 0.0000 | 0.0000 |
| **'NADPH_consume'** | 0.0009 | 0.0008 | 0.0006 | 0.0008 | 0.0006 | 0.0006 |
| **'DCW'** | 0.4062 | 0.3733 | 0.3041 | 0.3610 | 0.2713 | 0.2893 |
| **'GLK1-HXK1-2'** | 0.0139 | 0.0146 | 0.0132 | 0.0136 | 0.0153 | 0.0128 |
| **'PGI1'** | 0.0112 | 0.0122 | 0.0112 | 0.0116 | 0.0136 | 0.0109 |
| **'FBA1'** | 0.0122 | 0.0130 | 0.0119 | 0.0123 | 0.0142 | 0.0116 |
| **'TPI1'** | -0.0099 | -0.0111 | -0.0098 | -0.0107 | -0.0111 | -0.0094 |
| **'TDH1-2-3'** | 0.0225 | 0.0245 | 0.0221 | 0.0232 | 0.0255 | 0.0214 |
| **'PGK1'** | 0.0225 | 0.0245 | 0.0221 | 0.0232 | 0.0255 | 0.0214 |
| **'GMP1-2-3'** | 0.0223 | 0.0243 | 0.0219 | 0.0230 | 0.0254 | 0.0212 |
| **'ENO1-2'** | 0.0223 | 0.0243 | 0.0219 | 0.0230 | 0.0254 | 0.0212 |
| **'PYC1-2'** | 0.0040 | 0.0046 | 0.0046 | 0.0036 | 0.0050 | 0.0043 |
| **'ZWF1'** | 0.0016 | 0.0015 | 0.0012 | 0.0011 | 0.0010 | 0.0011 |
| **'RKI1'** | 0.0005 | 0.0005 | 0.0004 | 0.0004 | 0.0003 | 0.0004 |
| **'RPE1'** | 0.0010 | 0.0009 | 0.0007 | 0.0006 | 0.0006 | 0.0007 |
| **'TKL1-2_1'** | -0.0005 | -0.0005 | -0.0004 | -0.0004 | -0.0003 | -0.0004 |
| **'TKL1-2_2'** | -0.0004 | -0.0004 | -0.0003 | -0.0003 | -0.0003 | -0.0003 |
| **'TAL1'** | 0.0005 | 0.0005 | 0.0004 | 0.0004 | 0.0003 | 0.0004 |
| **'ACO1'** | -0.1786 | -0.2341 | -0.2568 | -0.3524 | -0.3671 | -0.2450 |
| **'FUM1'** | -80.5146 | -76.4444 | -78.8327 | -98.0087 | -102.069 | -77.7464 |
| **'FRDS1-OSM1'** | 116.9788 | 131.5838 | 136.2037 | 149.6442 | 149.9132 | 133.8191 |
| **'ADH1-2-4-5'** | 245.9242 | 259.3022 | 265.4342 | 312.5153 | 300.7887 | 264.2550 |
| **'ADH3_m'** | -245.908 | -259.283 | -265.418 | -312.497 | -300.769 | -264.240 |
| **'ALD4_m'** | -0.0004 | -0.0003 | -0.0003 | -0.0005 | -0.0002 | -0.0003 |
| **'LAT1_m'** | -0.0032 | -0.0031 | -0.0049 | -0.0015 | -0.0053 | -0.0047 |
| **'LPD1_m'** | 0.0032 | 0.0031 | 0.0049 | 0.0015 | 0.0053 | 0.0047 |
| **'LSC1-2_m'** | 0.0000 | 0.0000 | 0.0000 | 0.0000 | 0.0000 | 0.0000 |
| **'SDH1-2-4_m'** | 116.9791 | 131.5839 | 136.2050 | 149.6442 | 149.9147 | 133.8204 |
| **'FUM1_m'** | 80.5143 | 76.4443 | 78.8313 | 98.0087 | 102.0682 | 77.7451 |
| **'MDH1_m'** | -245.908 | -259.284 | -265.420 | -312.496 | -300.771 | -264.241 |
| **'ACO1_m'** | 0.1793 | 0.2345 | 0.2585 | 0.3529 | 0.3689 | 0.2467 |
| **'M_Ac-CoA'** | 0.0031 | 0.0030 | 0.0048 | 0.0014 | 0.0052 | 0.0046 |
| **'M_CO2'** | -0.0037 | -0.0036 | -0.0053 | -0.0018 | -0.0056 | -0.0051 |
| **Additional File 6 – Intracellular fluxes for metabolic network (cont’d)*** | | | | | | |
| **Reaction Name^†^** | **WTN** | **WTI** | **WTA** | **dN** | **dI** | **dA** |
| **'M_O2'** | 0.0043 | 0.0041 | 0.0072 | 0.0027 | 0.0072 | 0.0068 |
| **'M_FAD'** | 116.9788 | 131.5838 | 136.2037 | 149.6442 | 149.9132 | 133.8191 |
| **'M_FADH2'** | -116.978 | -131.583 | -136.203 | -149.644 | -149.913 | -133.819 |
| **'KGD2_m'** | 0.0000 | 0.0000 | 0.0000 | 0.0000 | 0.0000 | 0.0000 |
| **'M_Actal'** | -245.908 | -259.283 | -265.418 | -312.497 | -300.769 | -264.240 |
| **'M_Mal-SucDIC1'** | 332.9663 | 356.4625 | 367.2699 | 430.7749 | 423.3032 | 364.1307 |
| **'M_Ox-OxODC1-2'** | 0.0000 | 0.0000 | 0.0000 | 0.0000 | 0.0000 | 0.0000 |
| **'M_Cit-IcitCTP1'** | -0.1793 | -0.2345 | -0.2585 | -0.3529 | -0.3689 | -0.2467 |

*Fluxes reported as mol/gDCW/h.

^†^Yeast central carbon metabolic model used (Forst*er et* al, 2002). All nomenclature follows the published model.
