# Supplementary material for: Imbalance of heterologous protein folding and disulfide bond formation rates yields runaway oxidative stress
Source: BMC Biol. 2012 Mar 1;10:16. doi: 10.1186/1741-7007-10-16 (PMC3310788; doi:10.1186/1741-7007-10-16)
Supplement: Additional file 5 — Reporter TFs for WT protein secretion. Transcription factors activated by recombinant protein secretion in wild-type background. [file 1741-7007-10-16-S5.DOC]

## Additional File 5 – Reporter TFs for WT Protein Secretion

|  |  | **Table 1 - Reporter TFs for WT General Protein Secretion** | | | | |
| --- | --- | --- | --- | --- | --- | --- |
| **Reg.*** | | | **IP Reporter**  **P value** | **Amylase Reporter**  **P value** | **TF Name** | **TF Class** |
| ↑ | | | 3.04E-4 | 2.07E-05 | CIN5 | Oxidative/Osmotic Stress Response |
| ↑ | | | 0.004109 | 0.0051856 | XBP1 | Stress/Starvation Control of Cell Cycle |
| ↑ | | | 4.21E-4 | 9.16E-4 | HSF1 | Thermal Stress Response |
| ↑ | | | 0.009928 | 6.95E-05 | MSN2 | General Stress Response |
| **↑↓** | | | 0.006658 | 0.0460687 | DAL81 | Organic Nitrogen Degradation |
| ↑ | | | 0.036084 | 4.93E-05 | SOK2 | Cell Cycle |
| **↑↓** | | | 0.038552 | 7.70E-4 | NRG1 | Glucose Repression |
| ↑ | | | 0.042854 | 0.00791675 | SKN7 | Oxidative/Osmotic Stress Response |
| **↓** | | | 0.039561 | 0.0120536 | GAL3 | Galactose Utilization |
| **↓** | | | 0.041230 | 0.0346584 | HAC1 | Unfolded Protein Response |
|  |  | *Regulation – Targets of TF are up or down regulated | | | | |
|  | | |  |  |  |  |
|  |  | **Table 2 - Reporter TFs for WT Small Protein Secretion** | | | | |
| **Reg.*** | | | **IP Reporter**  **P value** | **Amylase Reporter**  **P value** | **TF Name** | **TF Class** |
| **↑** | | | 0.0003007 | 0.0966891 | MCM1 | Pheremone Response |
| **↑** | | | 3.65E-05 | 0.200884 | FKH2 | Cell Cycle |
| **↑** | | | 0.001804 | 0.469585 | MBP1 | Cell Cycle |
| **↑** | | | 0.004767 | 0.170754 | SWI6 | Cell Cycle |
| **↑** | | | 0.008535 | 0.0966345 | NDD1 | Cell Cycle |
| **↓** | | | 0.017158 | 0.128961 | CST6 | AFT/CREB TF - Non-optimal carbon source |
| **↑↓** | | | 0.024311 | 0.0650543 | STO1 | Overall mRNA control |
| **↑** | | | 0.038010 | 0.0755082 | MSN4 | Stress Response |
| **↓** | | | 0.038890 | 0.1561 | UPC2 | Sterol Biosynthesis |
| **↓** | | | 0.039610 | 0.117658 | STE12 | Invasive Growth |
|  |  | *Regulation – Targets of TF are up or down regulated | | | | |

|  |  | **Table 3 - Reporter TFs for WT Large Protein Secretion** | | | | |
| --- | --- | --- | --- | --- | --- | --- |
| **Reg.*** | | | **IP Reporter**  **P value** | **Amylase Reporter**  **P value** | **TF Name** | **TF Class** |
| ↑ | | | 0.0893439 | 0.0051132 | SKO1 | Oxidative/Osmotic Stress Response |
| ↑ | | | 0.778898 | 0.0096119 | AFT2 | Oxidative Stress |
| ↑ | | | 0.0966919 | 0.0215226 | MIG3 | General Stress Response |
| **↑↓** | | | 0.398792 | 0.0010358 | YAP7 | Unknown |
| **↓** | | | 0.661159 | 0.0111131 | BAS1 | Purine/Histidine Biosynthesis |
| ↑ | | | 0.75781 | 0.0200425 | AZF1 | Glucose Response |
| **↓** | | | 0.0606936 | 0.0208618 | PHO2 | Phosphate Metabolism |
| **↑↓** | | | 0.244058 | 0.0228582 | MOT3 | Oxygen/Ergosterol Synthesis |
| ↑ | | | 0.0593748 | 0.0239297 | HIR2 | Chromatin Silencing |
| **↑↓** | | | 0.293962 | 0.0253314 | CAD1 | Stress Response |
| ↑ | | | 0.290363 | 0.0297114 | UME6 | Nutritional Sensing for Myosis |
| ↑ | | | 0.0576075 | 0.0306253 | PHD1 | Invasive Growth |
| ↑ | | | 0.112671 | 0.0321627 | ARG80 | Arginine Biosynthesis |
| ↑ | | | 0.455592 | 0.0325519 | YAP6 | Osmotic Stress Response |
| ↑ | | | 0.209152 | 0.0335437 | MSN1 | Secreted Protein Synthesis / Osmotic Stress |
| **↓** | | | 0.710722 | 0.0354209 | THO2 | Overall Transcriptional Regulation |
| **↓** | | | 0.461552 | 0.0354986 | MET32 | Methionine Biosynthesis |
|  |  | *Regulation – Targets of TF are up or down regulated | | | | |
