# Supplementary material for: Imbalance of heterologous protein folding and disulfide bond formation rates yields runaway oxidative stress
Source: BMC Biol. 2012 Mar 1;10:16. doi: 10.1186/1741-7007-10-16 (PMC3310788; doi:10.1186/1741-7007-10-16)
Supplement: Additional file 6 — Reporter TFs for Δhac1 protein secretion. Transcription factors activated by recombinant protein secretion in Δhac1 background. [file 1741-7007-10-16-S6.DOC]

## Additional File 6 – Reporter TFs for Δ*hac1* Protein Secretion

|  | **Table 1 - Reporter TFs for Δ*hac1* General Protein Secretion** | | | | |
| --- | --- | --- | --- | --- | --- |
| **Reg.*** | | **IP Reporter**  **P value** | **Amylase Reporter**  **P value** | **TF Name** | **TF Class** |
| **↑↓** | | 0.0004362 | 0.0148005 | CAD1 | Stress Response |
| **↑** | | 3.53E-06 | 7.73E-06 | CIN5 | Oxidative/Osmotic Stress Response |
| **↓** | | 0.013459 | 0.0001312 | DAL81 | Organic Nitrogen Degradation |
| **↓** | | 0.005565 | 0.0085637 | GCN4 | Amino Acid Synthesis |
| **↑↓** | | 0.010984 | 0.0072941 | HAP1 | Oxygen-dependent Growth |
| **↑** | | 0.0006498 | 7.30E-05 | HSF1 | Thermal Stress Response |
| **↑** | | 0.0007429 | 0.0017741 | MSN2 | General Stress Response |
| **↓** | | 0.0300432 | 0.037044 | OTU1 | Deubiquination |
| **↑** | | 0.0140043 | 0.0022747 | PHD1 | Invasive Growth |
| **↑** | | 0.031297 | 0.019443 | RIM101 | Invasive Growth / Cell Wall Synthesis |
| **↓** | | 0.0443422 | 0.025154 | RTG3 | Glutamine Synthesis |
| **↑** | | 0.0093903 | 0.0070845 | SKN7 | Oxidative/Osmotic Stress Response |
| **↑** | | 0.007428 | 0.018743 | SNT2 | Amine Transporters |
| **↑** | | 6.36E-05 | 0.0001476 | SOK2 | Cell Cycle |
| **↑** | | 0.0261086 | 0.010241 | STO1 | mRNA Transport/Degradation |
| **↑** | | 0.0009199 | 0.029928 | XBP1 | Stress/Starvation Control of Cell Cycle |
| **↑↓** | | 0.0087531 | 0.005922 | YAP6 | Osmotic Stress Response |
| **↓** | | 0.0001468 | 3.49E-07 | YAP7 | Unknown |
|  | | *Regulation – Targets of TF are up or down regulated | | | |

|  | **Table 2 - Reporter TFs for Δ*hac1* Small** **Protein Secretion** | | | | |
| --- | --- | --- | --- | --- | --- |
| **Reg.*** | | **IP Reporter**  **P value** | **Amylase Reporter**  **P value** | **TF Name** | **TF Class** |
| **↓** | | 0.01734 | 0.38679 | ARG81 | Arginine Biosynthesis |
| **↓** | | 0.03317 | 0.09560 | AZF1 | Glucose Response |
| **↓** | | 3.55E-07 | 0.99906 | FHL1 | Ribosome Synthesis |
| **↑** | | 0.04787 | 0.21845 | GCR2 | Glycolytic Genes |
| **↑** | | 0.04734 | 0.13615 | MDS3 | Mating Response |
| **↑** | | 0.02823 | 0.47692 | OAF1 | Beta-oxidation and Peroxisome |
| **↓** | | 0.009135 | 0.05100 | PUT3 | Proline utilization |
| **↓** | | 6.91E-05 | 0.99435 | RAP1 | Overall Transcriptional Regulation |

|  | **Table 3 - Reporter TFs for Δ*hac1* Large Protein Secretion** | | | | |
| --- | --- | --- | --- | --- | --- |
| **Reg.*** | | **IP Reporter**  **P value** | **Amylase Reporter**  **P value** | **TF Name** | **TF Class** |
| **↓** | | 0.25700 | 0.038320 | ASH1 | Mating Type Switching |
| **↑** | | 0.05096 | 0.005858 | CUP9 | Peptide Transporters |
| **↑** | | 0.55850 | 0.013394 | FKH2 | Cell Cycle |
| **↑↓** | | 0.23086 | 0.009863 | HAC1 | Unfolded Protein Response |
| **↓** | | 0.13047 | 0.025260 | MTH1 | Cell Cycle |
| **↑** | | 0.30922 | 0.038716 | NDD1 | Cell Cycle |
| **↑** | | 0.06672 | 0.001027 | NRG1 | Glucose Repression |
| **↓** | | 0.31583 | 0.045270 | PDC2 | Glycolysis |
| **↑↓** | | 0.10573 | 0.037930 | PHO2 | Phosphate Metabolism |
| **↓** | | 0.23953 | 0.025888 | PHO4 | Phosphate Metabolism |
| **↑↓** | | 0.56574 | 0.041989 | RGT1 | Glucose Transporters |
| **↑↓** | | 0.05730 | 0.007673 | ROX1 | Oxygen-dependent Growth |
| **↓** | | 0.51566 | 0.048979 | SWI4 | Cell Cycle |
| **↓** | | 0.06492 | 0.000453 | YAP1 | Oxidative Stress |
