# Supplementary material for: Imbalance of heterologous protein folding and disulfide bond formation rates yields runaway oxidative stress
Source: BMC Biol. 2012 Mar 1;10:16. doi: 10.1186/1741-7007-10-16 (PMC3310788; doi:10.1186/1741-7007-10-16)
Supplement: Additional file 7 — Expression profiles for ribosomal proteins. mRNA concentrations for yeast ribosomal proteins as determined by DNA microarray. [file 1741-7007-10-16-S7.DOC]

## Additional File 7. –Ribosomal Protein Expression is downregulated with insulin expression in Δ*hac1* strain

While few genes for the ribosomal proteins exhibited a stringent P value, taken as a group, it is likely that the ribosomal proteins are upregulated in the *hac1* no protein (dN) state over the insulin state (dI). Note, the log_2_ ratio is dN/dI, therefore a positive value is downregulation in the dI strain.
