# Supplementary material for: Imbalance of heterologous protein folding and disulfide bond formation rates yields runaway oxidative stress
Source: BMC Biol. 2012 Mar 1;10:16. doi: 10.1186/1741-7007-10-16 (PMC3310788; doi:10.1186/1741-7007-10-16)
Supplement: Additional file 8 — Oligonucleotides used in this study. PCR primers used for cloning and validation. [file 1741-7007-10-16-S8.DOC]

| **Additional File 8 - Oligonucleotides used in this study** | |
| --- | --- |
| Primer | Sequence |
| KT007 | TATCTATACAAAAAACGGCAGACAATGCAG |
| KT008 | GGAGACAGAACAGTAGAAACCACTAAGCGA |
| LZH015 | TCTCACACTAGTATGAAATTGAAAACTGTTAGATCTG |
| LZH016 | GCCAGTCCGCAGGCGTTGCTTTTGGTTCACCTTCTTCTCTT |
| LZH018 | GCAACGCCTGCGGACT |
| LZH039 | TCATGGATCCCACTGTCCAATGC |
