# Supplementary material for: Imbalance of heterologous protein folding and disulfide bond formation rates yields runaway oxidative stress
Source: BMC Biol. 2012 Mar 1;10:16. doi: 10.1186/1741-7007-10-16 (PMC3310788; doi:10.1186/1741-7007-10-16)
Supplement: Additional file 9 — Synthesized insulin precursor DNA sequence. DNA sequence for insulin precursor used in this study. [file 1741-7007-10-16-S9.DOC]

## Additional File 9. Synthesized Insulin Precursor DNA Sequence

ACT AGT AAC AAA ATG AAA TTG AAA ACT GTT AGA TCT GCT GTT TTG TCT TCT TTG TTT GCT

1 --- --- --- +-- --- --- -+- --- --- --+ --- --- --- +-- --- --- -+- --- --- --+ 60

TGA TCA TTG TTT TAC TTT AAC TTT TGA CAA TCT AGA CGA CAA AAC AGA AGA AAC AAA CGA

Met Lys Leu Lys Thr Val Arg Ser Ala Val Leu Ser Ser Leu Phe Ala

TCT CAA GTT TTG GGT CAA CCA ATT GAT GAT ACT GAA TCT CAA ACT ACT TCT GTT AAT TTG

61 --- --- --- +-- --- --- -+- --- --- --+ --- --- --- +-- --- --- -+- --- --- --+ 120

AGA GTT CAA AAC CCA GTT GGT TAA CTA CTA TGA CTT AGA GTT TGA TGA AGA CAA TTA AAC

Ser Gln Val Leu Gly Gln Pro Ile Asp Asp Thr Glu Ser Gln Thr Thr Ser Val Asn Leu

ATG GCT GAT GAT ACT GAA TCT GCT TTT GCT ACT CAA ACT AAT TCT GGT GGT TTG GAT GTT

121 --- --- --- +-- --- --- -+- --- --- --+ --- --- --- +-- --- --- -+- --- --- --+ 180

TAC CGA CTA CTA TGA CTT AGA CGA AAA CGA TGA GTT TGA TTA AGA CCA CCA AAC CTA CAA

Met Ala Asp Asp Thr Glu Ser Ala Phe Ala Thr Gln Thr Asn Ser Gly Gly Leu Asp Val

GTT GGT TTG ATT TCT ATG GCT AAA AGA GAA GAA GGT GAA CCA AAA TTT GTT AAT CAA CAT

181 --- --- --- +-- --- --- -+- --- --- --+ --- --- --- +-- --- --- -+- --- --- --+ 240

CAA CCA AAC TAA AGA TAC CGA TTT TCT CTT CTT CCA CTT GGT TTT AAA CAA TTA GTT GTA

Val Gly Leu Ile Ser Met Ala Lys Arg Glu Glu Gly Glu Pro Lys Phe Val Asn Gln His

TTG TGT GGT TCT CAT TTG GTT GAA GCT TTG TAT TTG GTT TGT GGT GAA AGA GGT TTC TTT

241 --- --- --- +-- --- --- -+- --- --- --+ --- --- --- +-- --- --- -+- --- --- --+ 300

AAC ACA CCA AGA GTA AAC CAA CTT CGA AAC ATA AAC CAA ACA CCA CTT TCT CCA AAG AAA

Leu Cys Gly Ser His Leu Val Glu Ala Leu Tyr Leu Val Cys Gly Glu Arg Gly Phe Phe

TAC ACT CCA AAG GAA TGG AAG GGT ATC GTT GAA CAA TGT TGT ACT TCT ATC TGT TCT TTG

301 --- --- --- +-- --- --- -+- --- --- --+ --- --- --- +-- --- --- -+- --- --- --+ 360

ATG TGA GGT TTC CTT ACC TTC CCA TAG CAA CTT GTT ACA ACA TGA AGA TAG ACA AGA AAC

Tyr Thr Pro Lys Glu Trp Lys Gly Ile Val Glu Gln Cys Cys Thr Ser Ile Cys Ser Leu

TAC CAA TTG GAA AAT TAT TGT AAT TAA GTC GAC

361 --- --- --- +-- --- --- -+- --- --- --+ --- 393

ATG GTT AAC CTT TTA ATA ACA TTA ATT CAG CTG

Tyr Gln Leu Glu Asn Tyr Cys Asn ***

13 Start Codon

13-75 Pre sequence (from Yap3)

76-201 Pro sequence (TA57)

226-384 Insulin Precursor

Pre, pro, and insulin precursor amino acid sequence from

### Supplemental References

1. Kjeldsen T, Pettersson AF, & Hach M (1999) The role of leaders in intracellular transport and secretion of the insulin precursor in the yeast Saccharomyces cerevisiae. *Journal of Biotechnology* 75(2-3):195-208.
